# Matrine Inhibits the Wnt3a/β‐Catenin Signalling to Attenuate Pressure Overload‐Induced Atrial Remodelling and Vulnerability to Atrial Fibrillation

**DOI:** 10.1111/jcmm.70617

**Published:** 2025-05-23

**Authors:** Guoxin Zhang, Xue Dong, Boxuan Sun, Zijun Zhou, Yinli Xu, Yuting Huang, Shan Meng, Zijun Cao, Nana Qin, Yan Zhu, Liming Yu, Huishan Wang

**Affiliations:** ^1^ State Key Laboratory of Frigid Zone Cardiovascular Disease, Department of Cardiovascular Surgery General Hospital of Northern Theater Command Shenyang Liaoning China; ^2^ The Third Outpatient Department General Hospital of Northern Theater Command Shenyang Liaoning China; ^3^ Jinzhou Medical University Jinzhou Liaoning China; ^4^ Liaoning University of Traditional Chinese Medicine Shenyang Liaoning China

**Keywords:** atrial fibrillation, atrial remodelling, matrine, Wnt3a, β‐Catenin

## Abstract

Atrial fibrillation (AF) is closely associated with atrial electrical and structural remodelling, yet effective drug strategies remain limited. Matrine (MAT), the active compound in *Sophora flavescens* , has shown anti‐AF effects, but its mechanisms are unclear. This study explored MAT's impact on pressure overload‐induced AF using clinical samples, bioinformatics, network pharmacology and murine models, focusing on the canonical Wnt signalling. A murine pressure overload model was established via transverse aortic constriction (TAC) surgery for 4 weeks. Programmed electrical stimulation, langendorff perfusion, echocardiography, Masson's trichrome staining and western blotting were used to evaluate the potential effects and mechanisms of MAT. The results demonstrated that TAC‐induced atrial electrical and structural remodelling significantly increased susceptibility to AF in mice while also up‐regulated atrial Wnt3a/β‐catenin signalling as well as markers for remodelling and inflammation, which were partially supported by clinical samples. MAT dose‐dependently mitigated atrial structural and electrical remodelling. Furthermore, MAT intervention inhibited Wnt3a/β‐catenin signalling. However, co‐administration of SKL2001, a Wnt/β‐catenin agonist, counteracted MAT's benefits. The overall findings suggest that MAT treatment may serve as a potential therapeutic approach for inhibiting TAC‐induced atrial electrical and structural remodelling by suppressing Wnt3a/β‐catenin signalling pathways, thereby reducing susceptibility to AF.

**Abbreviations**
AF: atrial fibrillation
BCA: bicinchoninic acid
BP: biological process
BSA: bovine serum albumin
COL1A1: collagen I
COL3A1: collagen III
Cx40: connexin‐40
Cx43: connexin‐43
DEGs: differentially expressed genes
Dvl: Dishevelled
ECGs: electrocardiograms
GO: gene ontology
GSEA: gene set enrichment analysis
GSK3β: glycogen synthetase 3β
HF: heart failure
IL‐1β: interleukin‐1β
IL‐6: interleukin‐6
KEGG: Kyoto Encyclopedia of Genes and Genomes
LA: left atrial
LVEF: left ventricular ejection fraction
LVFS: left ventricular fractional shortening
LVIDd: left ventricular internal diameter at end‐diastolic
LVIDs: left ventricular internal diameter at end‐systolic
LVPWd: left ventricular posterior wall diameter
MAT: matrine
MMP2: matrix metallopeptidase 2
MMP9: matrix metallopeptidase 9
PPI: protein–protein interaction
SDS‐PAGE: sodium dodecyl sulphate‐polyacrylamide
TAC: transversal aortic contraction
TGF‐β: transforming growth factor β
TNF‐α: tumour necrosis factor α
WGA: wheat germ agglutinin
α‐SMA: α‐smooth muscle actin

## 1 Introduction

Despite extensive efforts to comprehend its pathophysiology and develop effective treatments, the incidence of atrial fibrillation (AF) continues to rise annually [1]. Epidemiological studies have established a close association between AF and factors such as hypertension, diabetes and heart failure (HF) [2]. The occurrence of AF exhibits an intricate association with atrial structure and electrophysiological remodelling [3]. In a clinical study, researchers observed that patients with persistent AF exhibited elevated left atrial (LA) pressure, leading to structural remodelling of the atria and significantly augmenting the complexity associated with treating AF [4]. However, there are limited therapeutic approaches against AF associated with pressure overload.

The Wnt signalling pathway is highly conserved throughout evolution and plays a pivotal role in diverse biological species [5]. Wnt signalling pathways can be categorised into typical and atypical pathways. The canonical Wnt pathway relies on β‐catenin‐mediated transcription of downstream target genes, whereas the non‐canonical Wnt pathways are β‐catenin independent and encompass calcium‐dependent (Wnt/Ca^2+^) pathways as well as planar cell polarity (Wnt/PCP) pathways [6]. In the normal adult heart, Wnt signalling remains quiescent. However, during heart injury‐induced remodelling, Wnt signalling is reactivated, thereby promoting tissue fibrosis and inflammatory changes [7]. Several studies have reported that Wnt/β‐catenin signalling not only promotes fibroblast aggregation but also exhibits synergistic effects with TGF‐β [8, 9]. Furthermore, through crosstalk with NF‐κB, Wnt/β‐catenin signalling enhances the production of pro‐inflammatory cytokines [10]. The Wnt3a protein is widely recognised as a prototypical signalling molecule within the Wnt family. The research findings indicate that activation of the Wnt3a/β‐catenin signalling pathway exerts a profound influence on atrial fibroblast transforming into myofibroblasts [11]. Nonetheless, the precise role of the Wnt3a/β‐catenin signalling pathway in pressure overload‐associated AF remains elusive.

Matrine (MAT, C_15_H_24_N_2_O) is a significant quinolizidine alkaloid derived from the roots of the traditional Chinese herb *Sophora flavescens* . Studies have demonstrated that MAT possesses anti‐inflammatory, anti‐fibrotic and antioxidant properties, which can aid in the prevention and treatment of a variety of cardiovascular diseases [12]. Previous research has demonstrated that MAT can effectively prevent myocardial hypertrophy and interstitial fibrosis induced by pressure overload following abdominal aortic coarctation in rats [13]. Notably, recent studies have revealed that MAT can inhibit cardiac remodelling and pathological fibrosis through the regulation of the RPS5/p38 signalling pathway, potentially benefiting the prevention and treatment of heart failure and arrhythmia [14]. Furthermore, Zhou et al. highlighted its potential as an anti‐arrhythmic agent by elucidating its impact on atrial ion channels in mice [15]. Nevertheless, the influence of MAT on pressure overload‐associated atrial remodelling and the progression of AF remains to be elucidated.

The objective of this study was to examine the potential role of canonical Wnt signalling in pressure overload‐induced AF and atrial remodelling and to further explore the impact of MAT treatment.

## 2 Materials and Methods

### 2.1 RNA Sequencing and Data Analysis

RNA sequencing was conducted according to the protocols described in our previous publications [16, 17]. Briefly, the mice were humanely euthanised, after which the atrial samples were immediately excised and rinsed. Total RNA was extracted, and an RNA library was prepared. The resulting libraries were then sequenced on the Illumina NovaSeq X system (Berry Genomics, Beijing, China). The differentially expressed genes (DEGs) were screened by R software (2023.06.0 Build 421). Genes with a *p*‐value < 0.05 and absolute Log fold change (LogFC) > 0.585 were considered differentially expressed between the Sham and TAC groups. For comparison between the TAC and TAC + MAT groups, a less stringent threshold of |LogFC| > 0.263 was applied to detect subtle changes. Visualisation of the heatmap was accomplished with TBtools‐II software (v2.119). The Gene Ontology (GO) enrichment analysis was carried out via the Metascape bioinformatics tool (http://metascape.org, v3.5.20240101), and the findings were graphically represented using the SRplot platform [18].

### 2.2 Network Construction

The targets for MAT were retrieved from the TCMSP (https://old.tcmsp‐e.com/tcmsp.php) and PharmMapper (http://lilab‐ecust.cn/pharmmapper/) databases. Subsequently, the UniProt database (https://www.uniprot.org/) was utilised to standardise these targets. A keyword search for “atrial fibrillation” was conducted in GeneCards, OMIM and DrugBank databases to identify associated targets. Subsequently, a Venn diagram was constructed to screen targets for MAT intervention in AF. To establish a protein–protein interaction (PPI) network among these intersecting targets, STRING (https://string‐db.org/) was employed. Furthermore, Cytoscape 3.9.1 software was utilised to extract core targets and construct an “active ingredient‐target‐pathway” network. Topological analysis using the Network Analysis function provided degree value information.

### 2.3 Human Atrial Samples

Right atrial tissue samples were collected from 12 male patients diagnosed with valvular heart disease, with 6 of them presenting with AF. The demographic characteristics of these patients are detailed in Table S1. Exclusion criteria included individuals over the age of 70, severe infectious diseases, significant hepatic or renal impairment and malignant neoplasms. Prior to enrolment, all participants underwent a 12‐lead electrocardiogram and were categorised into either sinus rhythm or atrial fibrillation groups. Informed consent was obtained from each patient. All experimental protocols were approved by the Clinical Medical Research Ethics Committee of the Northern Theatre General Hospital (No. 2023‐005) and were performed in accordance with the Declaration of Helsinki.

### 2.4 Animals and Treatments

This study strictly adhered to the Institutional Animal Care and Use Guidelines and received approval from the Ethics Committee of the General Hospital of the Northern Theatre Command (No. 2024‐05). Male C57/BL6 mice (9 weeks of age) obtained from Huafukang Biotechnology Co., LTD (Beijing, China) were fed normal food and water for 1 week prior to the experiment. The study was conducted in two sequential phases. In the first phase, mice were randomly assigned to four groups (15 animals per group): Sham group, TAC group, TAC + 50 mg/kg MAT group (TAC + L‐MAT) and TAC + 100 mg/kg MAT group (TAC + H‐MAT). The dose of MAT used in the second phase was determined based on the optimal dose identified in the first phase of the experiment. In the second phase, mice were randomly divided into five groups (15 animals per group): Sham group, TAC group, TAC + 10 mg/kg SKL2001 group (TAC + SKL2001), TAC + MAT group (TAC + MAT) and TAC + 10 mg/kg SKL2001 + MAT group (TAC + SKL2001 + MAT). A TAC model was constructed using the previously described method [19]. Briefly, tracheal intubation was performed under 1%–2% isoflurane anaesthesia. After a midline sternotomy, a 6–0 silk suture was secured around a 27‐gauge needle positioned near the aorta distal to the brachiocephalic artery. Finally, the sternum and skin were closed. Animals in the sham surgery group underwent a similar procedure without TAC induction (Figures 2A and 5A). MAT (purity > 98%) was purchased from MCE biotechnology (#HY‐N0164). The dose of MAT and SKL2001 was chosen based on the preliminary experiment and previous publication [20, 21]. Mice in the Sham and TAC groups were administered equal volumes of water via intragastric delivery for a duration of 4 weeks.

### 2.5 Echocardiography

In accordance with the methodology outlined in our previous study [22], the left cardiac system in mice was assessed using a Doppler ultrasound imaging system (D700, VINNO, Suzhou, China). Briefly, following anaesthesia administration, the mice were placed in a supine position for the assessment of left cardiac function through the recording of parasternal long‐axis movement. Dedicated software was then employed to compute various parameters, including left ventricular posterior wall diameter (LVPWd), left ventricular end‐diastolic diameter (LVIDd), left ventricular ejection fraction (LVEF), left ventricular fractional shortening (LVFS), left atrial diameter (LAD) and left atrial area.

### 2.6 Programmed Intracardiac Stimulation

To investigate the susceptibility to AF, we conducted cardiac burst pacing using a methodology described in our previous study [23]. Briefly, the mice were placed in a supine position and anaesthetised. The 1‐1‐French octupole catheter (Transonic Scisense Inc., Ontario, Canada) was inserted into the right atrium via the right jugular vein and connected to the RA‐834 recorder (iWorx System Inc., Dover, NH, USA). PowerLab monitoring system and LabChart v7 software (ADInstruments, Castle Hill, Australia) were used to observe the cardiac activity of mice. Atrial arrhythmias were induced by 25 Hz 3‐s burst pacing. AF is defined as a rapid or irregular atrial rhythm that lasts at least 1 s. Successful AF induction necessitated at least two rapid pacings within three consecutive stimuli.

### 2.7 Electrophysiological Mapping

The electrophysiological mapping of the left atrium was conducted in accordance with our previously described methodology [16, 22, 24]. In brief, the mice were euthanised under anaesthesia, and their hearts were subsequently excised. The mice's hearts were cannulated through the aorta and connected to the Langendorff system, with Tyrode's solution perfused at a constant temperature of 37.0°C and a flow rate of 2–5 mL/min. Epicardial conduction signals were recorded using a multi‐electrode array consisting of a 64‐channel multi‐electrode array (MappingLab Ltd., Oxford, UK) placed on the surface of the left atrium while data acquisition was performed utilising EMapRecord software 5.0 (MappingLab Ltd., Oxford, UK). Subsequently, EMapScope software 5.0 (MappingLab Ltd., Oxford, UK) was employed for analysing parameters including average conduction velocity as well as absolute and relative inhomogeneity indices for each experimental group.

### 2.8 Masson's Trichrome Staining

Masson's trichrome staining was performed as described previously [24]. The hearts of mice in each group were fixed overnight in a 4% paraformaldehyde solution (pH 7.2, 158,127, Sigma‐Aldrich, USA), followed by dehydration and embedding in paraffin. Subsequently, the paraffin‐embedded samples were sectioned into 6 μm slices and stained with Masson's trichrome according to the manufacturer's protocol (abs9348, Absin, Shanghai, China). The fibrotic area was quantified for each section using Image‐Pro Plus 6.0 (Media Cybernetics, USA).

### 2.9 Immunofluorescence Staining

Immunofluorescence staining was employed to assess the expressions of atrial connexin 40 (Cx40), connexin 43 (Cx43), α‐SMA, Wnt3a and active β‐catenin as described previously [16]. Atrial tissue sections were prepared at a thickness of 5 μm and treated with 0.2% Triton X‐100 for 15 min followed by blocking (10% BSA, 37°C, 1.5 h). Subsequently, the tissue sections were incubated overnight at 4°C with the specified primary antibody. Information on the primary antibodies used was listed in Table S2. Nuclei were stained with DAPI (Beyotime Biotechnology, Shanghai, China) for 10 min, and cell membranes were stained with wheat germ agglutinin (WGA, 10 μg/mL, W11261, Invitrogen, Carlsbad, CA, USA). Fluorescence levels were subsequently captured using a Nikon C2 Plus confocal microscope (Nikon, Tokyo, Japan). The resulting images were analysed using ImageJ software.

### 2.10 Cytoplasmic and Nuclear Protein Extraction and Western Blot Analysis

Western blot experiment was conducted as described in the previous report [17]. Cytoplasmic and nucleoprotein extraction was conducted using a commercial kit (P0028, Beyotime Biotechnology, Shanghai, China) following the manufacturer's instructions. The protein from mice atrial tissue was extracted, and the protein concentration was determined using the BCA protein concentration detection kit (P0010S, Beyotime Biotechnology, Shanghai, China). Then, the protein samples were separated by 8%–12% SDS‐PAGE and subsequently transferred onto PVDF membranes for western blotting. The membranes were sealed with 5% skim milk (A600669, Sangon Biotech, Shanghai, China) for a duration of 2 h, followed by an overnight incubation at 4°C with the primary antibody. Information on the primary antibodies used was listed in Table S2. The membranes were subsequently washed and incubated with a horseradish peroxidase‐conjugated secondary antibody at ambient temperature for a duration of 2 h. Imprints were detected through enhanced chemiluminescence and scanned using a chemiluminescence system (Tanon Technology, Shanghai, China). The obtained images were analysed with Image J software (Table S3).

### 2.11 Data Analysis and Statistics

The data were analysed using GraphPad Prism software (Version 9.0, San Diego, CA, USA) and presented as mean ± SEM. Student's *t*‐test was used to compare the two groups. Groups were compared using an unpaired 2‐tailed *t*‐test, one‐way or two‐way ANOVA with Bonferroni post hoc test [25]. AF durations were assessed using a Mann–Whitney *U* test with Dunn's multiple comparison test as the data were not normally distributed (D'Agostino and Pearson omnibus normality test). To analyse the data of AF inducibility, the Fisher exact test was employed [26]. Statistical significance was defined as *p* < 0.05.

## 3 Results

### 3.1 Bioinformatics Analysis and Clinical Evidence Revealed the Potential Involvement of Canonical Wnt Signalling in the Pathogenesis of AF

**FIGURE 1:**
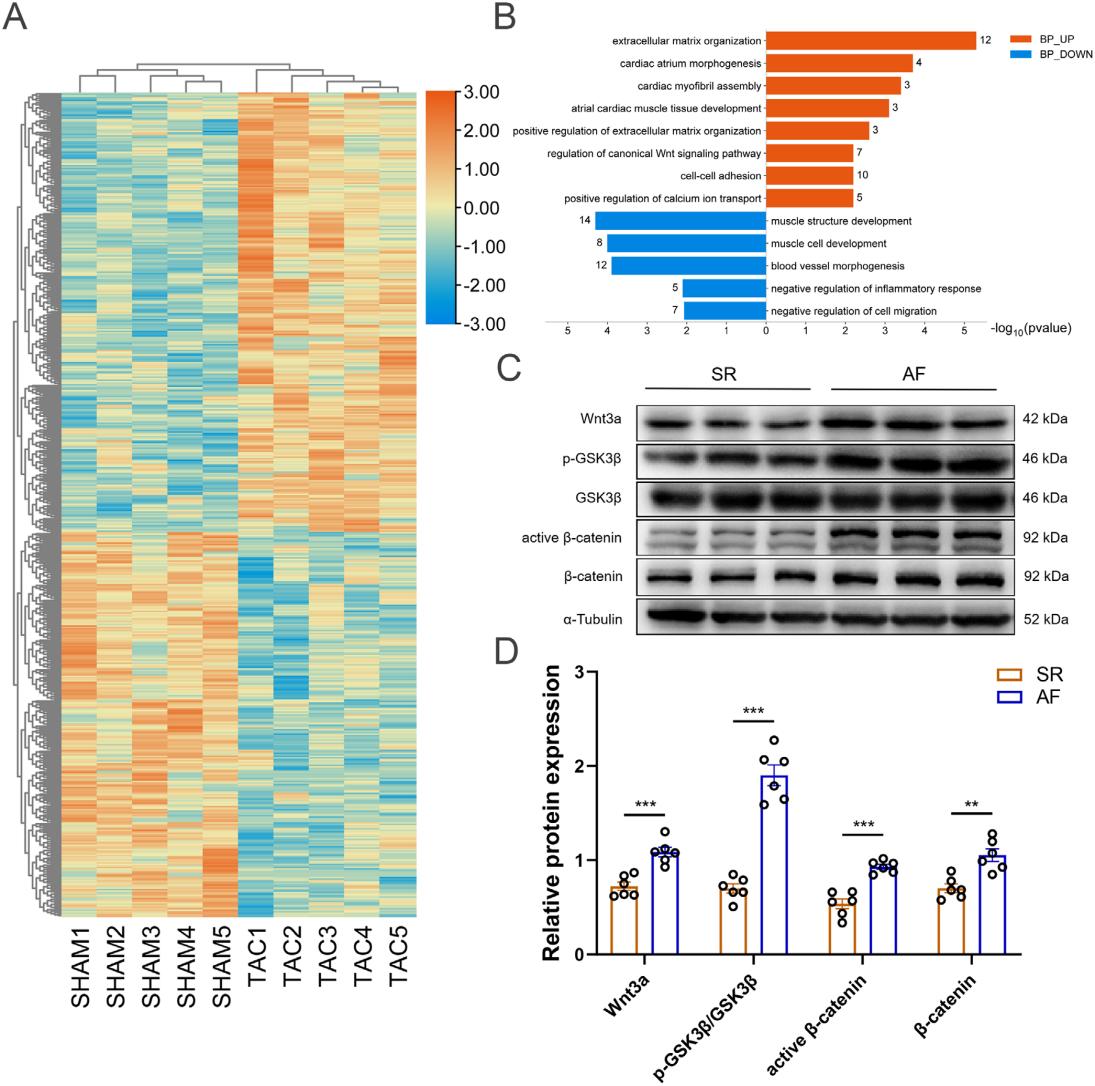
Activation of the Wnt3a/β‐catenin signalling pathway in the atrial tissue of patients diagnosed with AF. (A) Heatmap showing 297 up‐regulated and 256 down‐regulated DEGs in the TAC group versus the Sham group. (B) GO enrichment results including BP (*n* = 5). (C, D) Representative western blot images and relative atrial expression of Wnt3a, p‐GSK3β, active β‐catenin, β‐catenin (*n* = 6). Data were expressed as mean ± SEM. **p < 0.01, ***p < 0.001.

To elucidate the potential mechanisms underlying AF genesis, we performed bulk RNA sequencing followed by GO analysis on atrial tissue samples obtained from mice. Here, 556 DEGs were identified with 297 up‐regulated and 259 down‐regulated (Figure 1A). Subsequent GO analysis revealed that the up‐regulated DEGs were mainly enriched in extracellular matrix organisation, cardiac atrium morphogenesis, cardiac myofibril assembly, atrial cardiac muscle tissue development and, importantly, regulation of the canonical Wnt signalling pathway (Figure 1B). These data suggested that the abnormality of extracellular matrix organisation, inflammatory response and, critically, aberrant regulation of the canonical Wnt signalling pathway might contribute to TAC surgery‐associated AF. The Wnt3a‐mediated canonical Wnt pathway within the Wnt family has been extensively investigated in cardiovascular disease. The Wnt3a/β‐catenin signalling pathway plays a pivotal role in cardiac remodelling, cardiac fibrosis and apoptosis [27, 28]. Therefore, we further validated the aforementioned findings using human right atrial appendage tissue samples. Western blot analysis revealed significant up‐regulated atrial Wnt3a and the downstream Wnt signalling molecules (Figure 1C,D). Taken together, these data indicate that Wnt3a and the canonical Wnt pathway are possibly involved in TAC‐induced atrial damage.

### 3.2 MAT Ameliorated Stress Overload‐Induced Cardiac Dysfunction, Atrial Dilatation and Atrial Fibrosis in Mice

**FIGURE 2:**
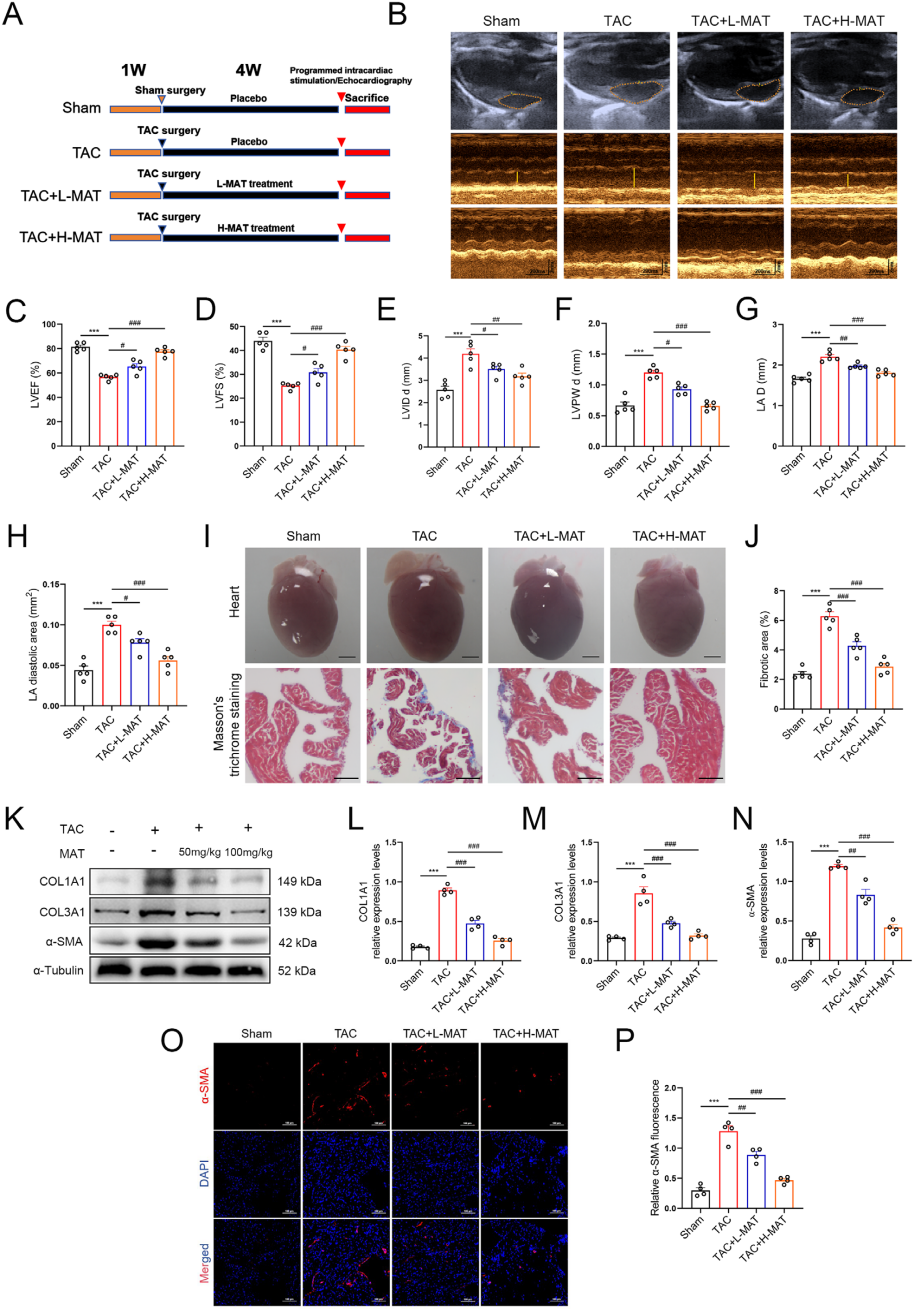
Potential of MAT to ameliorate atrial remodelling and mitigate tissue fibrosis induced by TAC. (A) Grouping of experimental animals and schematic diagram of the experiment. (B) Representative echocardiographic images of the atria and ventricle. (C–H) Echocardiographic parameters including left ventricular ejection fraction (LVEF), left ventricular shortening fraction (LVFS), left ventricular end‐diastolic diameter (LVIDd), left ventricular posterior wall diameter (LVPWd), left atrial diameter (LAD) and left atrial diastolic area (*n* = 5). (I) Overall cardiac morphology and representative Masson's trichromatic staining images (upper bar = 2 mm, lower bar = 100 μm). (J) Percentage of the total area with fibrosis (*n* = 5). (K–N) Representative western blot images and relative expression of COL1A1, COL3A1 and α‐SMA in each group of atria (*n* = 4). (O, P) Relative immunofluorescence intensity of α‐SMA (*n* = 4). Data were expressed as mean ± SEM. ***p < 0.001, versus Sham group; ^#^ *p* < 0.05, ^##^ *p* < 0.01, ^###^ *p* < 0.001, versus TAC group.

To assess the impact of MAT on cardiac function and atrial structure in TAC‐injured mice, echocardiographic analysis was conducted. The results indicated that the TAC‐induced mice group displayed heart enlargement and impaired cardiac function. However, these adverse effects were mitigated following MAT intervention (Figure 2B–H). Moreover, Masson's trichromatic staining revealed a significant reduction in atrial fibrosis after MAT intervention, showing a dose‐dependent pattern (Figure 2I,J). Additionally, to assess the potential therapeutic effects of MAT therapy on atrial fibrosis in TAC mice, we quantified the expression levels of COL1A1, COL3A1 and α‐SMA protein in atrial tissue. Western blot analysis (Figure 2K–N) and immunofluorescence staining (Figure 2O,P) demonstrated a notable decrease in COL1A1, COL3A1 and α‐SMA levels, as well as α‐SMA fluorescence intensity, following MAT intervention compared to the TAC group. These results indicate that MAT exerts a significant inhibitory effect on TAC‐induced cardiac dysfunction and atrial remodelling in mice.

### 3.3 MAT Attenuated Stress Overload‐Induced Atrial Electrical Remodelling and Connexin Remodelling in Mice

**FIGURE 3:**
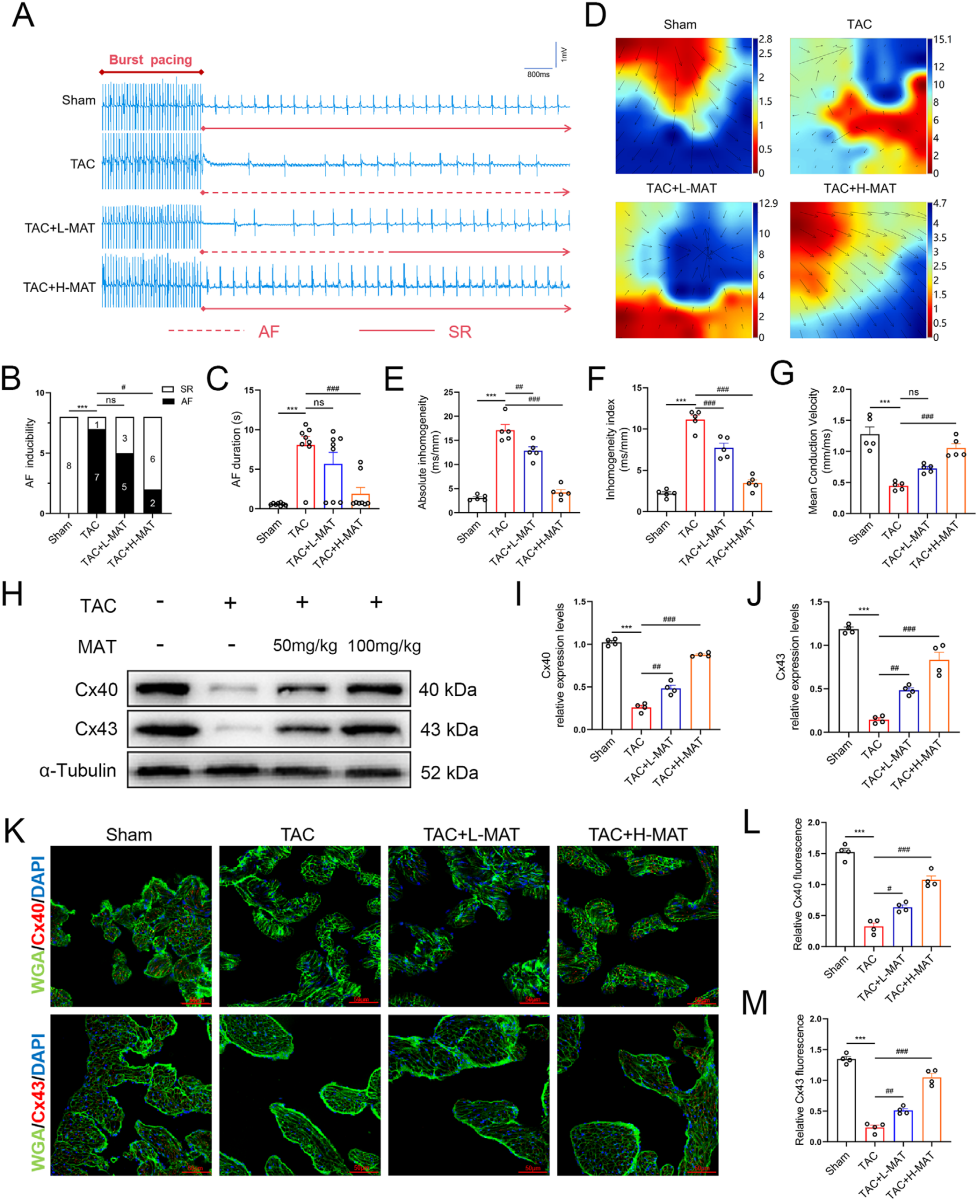
Potential of MAT to ameliorate atrial remodelling induced by TAC. (A) Representative ECG traces following burst pacing in the right atrium. The dashed line represents the atrial fibrillation rhythm. (B, C) Summary of AF induction and AF duration in each group (*n* = 8). (D) Langendorff electrophysiological mapping images. The bars represent the duration from the initial to the final measurement within one heartbeat (*n* = 5). (E–G) Summary of left atrial activation patterns. EMapScope 5.0 software was used to calculate the mean conduction velocity, absolute inhomogeneity and inhomogeneity index of the left atrium (*n* = 5). (H–J) Representative western blot images and relative atrial expression of Cx40 and Cx43 (*n* = 4). (K–M) WGA (green), Cx40 and Cx43 (red) and DAPI (blue) immunofluorescence stain representation and Cx40 and Cx43 relative immunofluorescence intensity (*n* = 4). Data were expressed as mean ± SEM. ***p < 0.001, versus Sham group; ^ns^ *p* > 0.05, ^#^ *p* < 0.05, ^##^ *p* < 0.01, ^###^ *p* < 0.001, versus TAC group.

Subsequently, we examined the effects of MAT on atrial electrical remodelling in mouse models induced by TAC. In the TAC group, the susceptibility to AF was markedly enhanced, and the duration of AF episodes was significantly prolonged. However, after MAT intervention, both the incidence and duration of AF were substantially reduced (Figure 3A–C). Furthermore, the results from the Langendorff cardiac perfusion system demonstrated that, compared to the Sham group, the atrial electrical conduction velocity was reduced, conduction heterogeneity was enhanced and the heterogeneity index was elevated in the TAC group. Notably, administration of MAT improved both atrial electrical conduction velocity and homogeneity indices (Figure 3D–G). Connexins are gap junction channels crucial for maintaining the electrophysiological functions of cardiomyocytes. We analysed Cx40 and Cx43 levels in atrial tissues through western blot analysis (Figure 3H–J) and immunofluorescence staining (Figure 3K–M), revealing enhanced connexin expression following MAT intervention, particularly with high concentrations. These findings suggest that TAC‐induced atrial electrical remodelling heightens susceptibility to AF, whereas MAT attenuates such remodelling and reduces vulnerability to AF.

### 3.4 MAT Effectively Inhibited Wnt3a/β‐Catenin Signalling Pathway, Attenuated Atrial Inflammation and Alleviated Atrial Fibrosis

**FIGURE 4:**
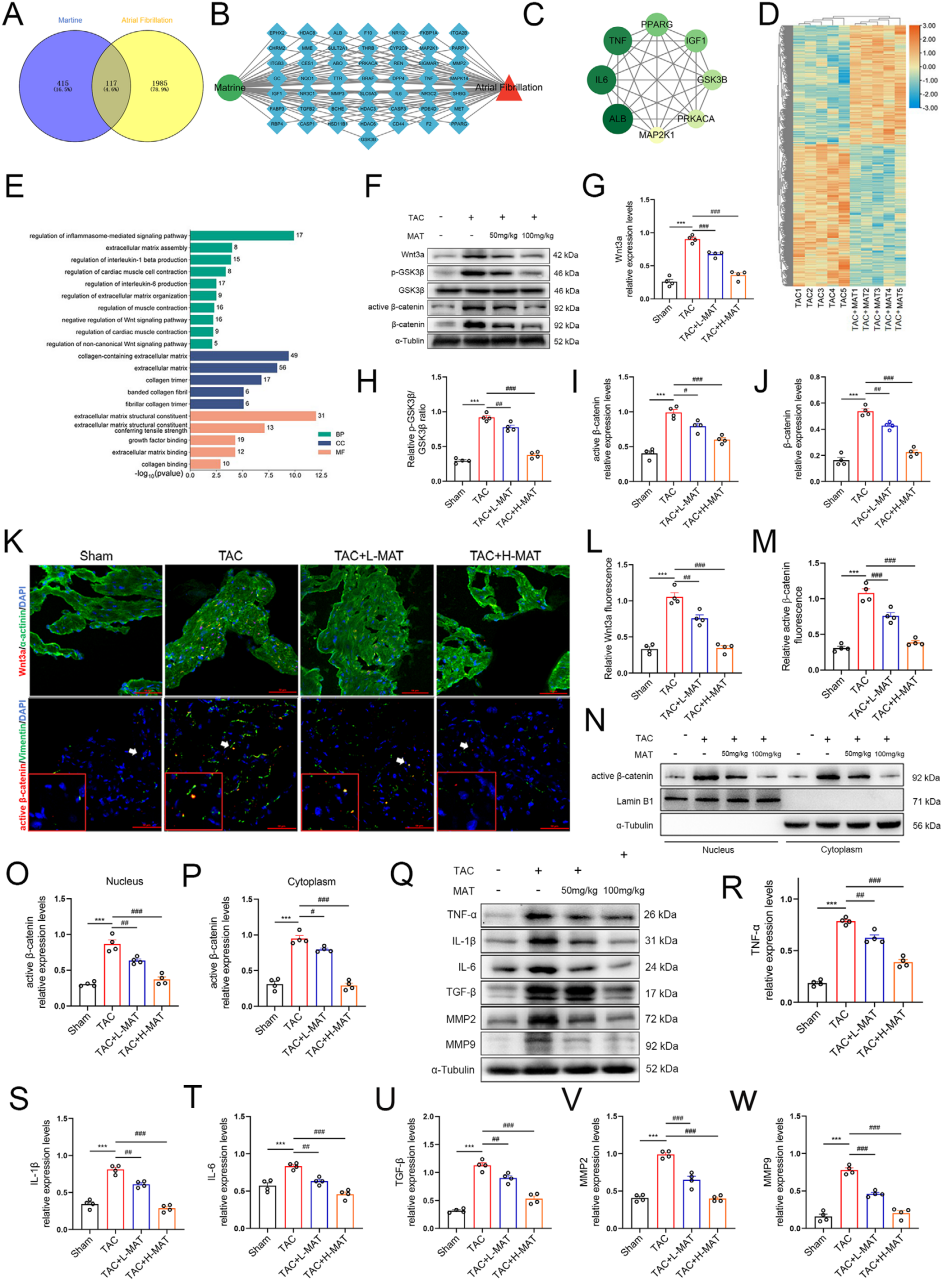
Effects of MAT administration on Wnt3a/β‐catenin signalling and atrial inflammation and fibrosis in mice subjected to TAC. (A) Venn diagram of the Matrine targets and AF targets. (B, C) MAT component‐target‐pathway network diagram and the key targets screened out. (D) Heatmap showing 593 up‐regulated and 706 down‐regulated DEGs in the TAC + MAT group versus the TAC group. (E) GO enrichment results including BP, CC, MF (*n* = 5). (F–J) Representative western blot images and relative atrial expression of Wnt3a, p‐GSK3β, active β‐catenin and β‐catenin (*n* = 4). (K–M) α‐actinin/Vimentin (green), Wnt3a/active β‐catenin (red) and DAPI (blue) immunofluorescence stain representation and Wnt3a/active β‐catenin relative immunofluorescence intensity (*n* = 4). (N–P) Representative western blot images and quantification of active β‐catenin expression in the atrial nucleus or cytoplasm (*n* = 4). (Q–W) Representative western blot images and relative atrial expression of TNF‐α, IL‐1β, IL‐6, TGF‐β, MMP2 and MM9 (*n* = 4). Data were expressed as mean ± SEM. ***p < 0.001, versus Sham group; ^#^ *p* < 0.05, ^##^ *p* < 0.01, ^###^ *p* < 0.001, versus TAC group.

To identify potential targets for MAT treatment of AF, we systematically screened 117 potentially relevant targets (Figure 4A). Through the analysis and screening of the obtained protein–protein interaction (PPI) network, eight core targets were identified: ALB, IL‐6, TNF, PPARG, IGF1 and GSK3β. Among these, GSK3β serves as a critical negative regulator of the Wnt3a/β‐catenin pathway. Its activity status directly governs the stability of β‐catenin, thereby influencing the progression of myocardial fibrosis and electrical remodelling (Figure 4B,C). To gain deeper insights into the pharmacological effects of MAT, we performed RNA sequencing on both the TAC‐induced injury group and the MAT‐treated group. The results were presented in Figure 4D, showing a heatmap of 1299 DEGs. Next, we conducted further GO analysis and found that biological processes including regulation of inflammasome‐mediated signalling pathway, regulation of interleukin‐1 beta production, regulation of cardiac muscle cell contraction, regulation of interleukin‐6 production and negative regulation of the Wnt signalling pathway were enriched (Figure 4E). Based on these findings, we further investigated the Wnt3a‐mediated canonical signalling pathway. Western blotting analysis (Figure 4F–J) revealed activation of this pathway in the TAC group, characterised by significantly increased levels of Wnt3a, active β‐catenin, β‐catenin and p‐GSK3β compared to the Sham group. Conversely, GSK3β levels were decreased (Figure 4J). Immunofluorescence staining for Wnt3a and active β‐catenin yielded similar findings (Figure 4K–M). Quantification of active β‐catenin levels in both the nucleus and cytoplasm demonstrated a significantly higher abundance in the TAC group compared to the Sham group (Figure 4N–P). Notably, MAT dose‐dependently inhibited the Wnt3a/β‐catenin signalling pathway when compared to the TAC group. Furthermore, western blotting tests were conducted according to the results of network pharmacology and RNA sequencing analysis. The results showed that compared with the Sham group, the expression levels of TGF‐β, MMP2, MMP9, TNF‐α, IL‐1β and IL‐6 were all increased in the TAC group, which was reversed by MAT intervention (compared to the TAC group, Figure 4Q–W). These findings suggest that MAT may modulate TAC‐induced atrial fibrosis and inflammation through inhibition of the Wnt3a/β‐catenin signalling pathway.

### 3.5 The Protective Effect of MAT on Atrial Structure Was Diminished by the Activation of the β‐Catenin Signalling

**FIGURE 5:**
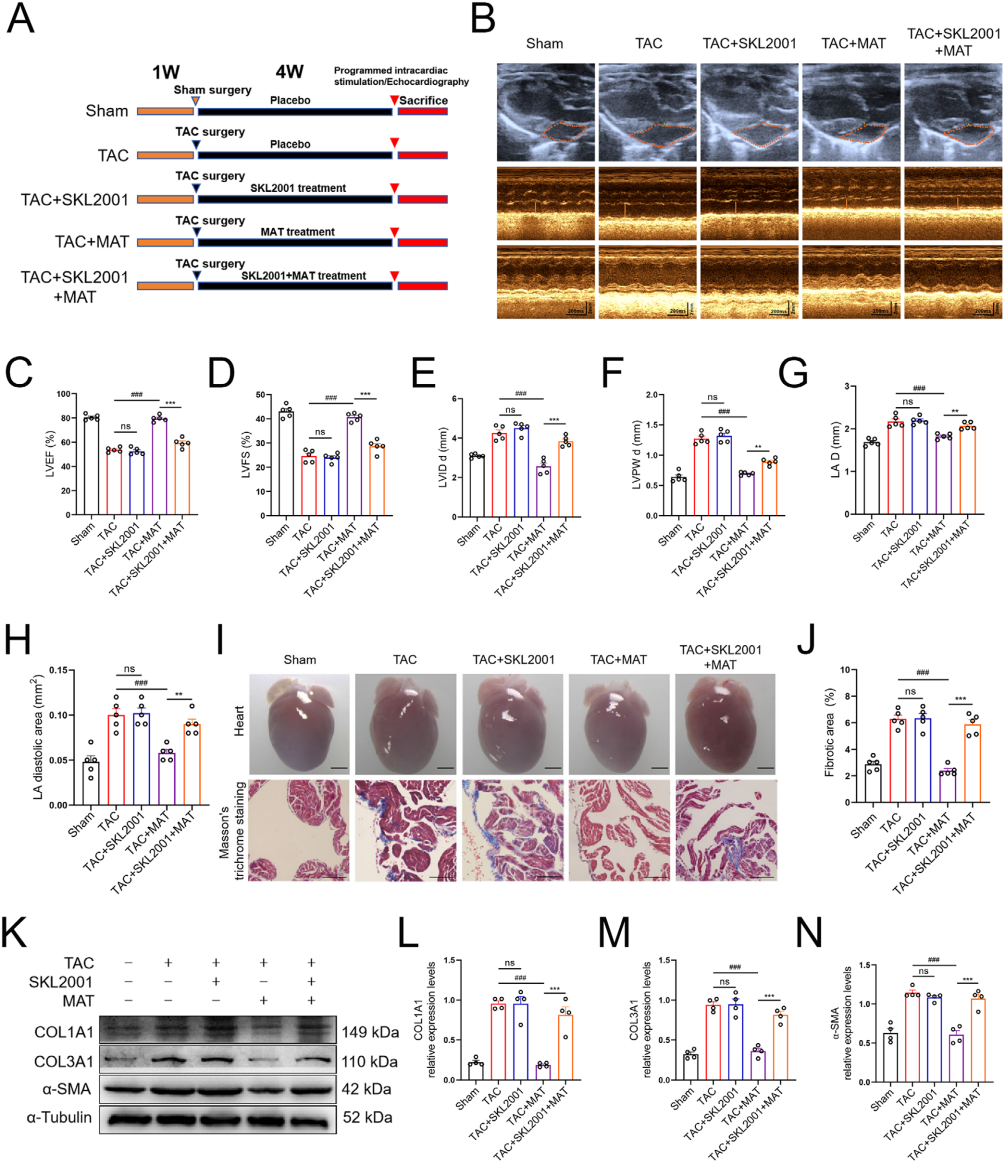
Reversal of the beneficial effect of MAT on atrial remodelling in TAC mice by activation of β‐catenin. (A) Grouping of experimental animals and schematic diagram of the experiment. (B) Representative echocardiographic images of the atria and ventricle. (C–H) Echocardiographic parameters including left ventricular ejection fraction (LVEF), left ventricular shortening fraction (LVFS), left ventricular end‐diastolic diameter (LVIDd), left ventricular posterior wall diameter (LVPWd), left atrial diameter (LAD) and left atrial diastolic area (*n* = 5). (I) Overall cardiac morphology and representative Masson's trichromatic staining images (upper bar = 2 mm, lower bar = 100 μm). (J) Percentage of the total area with fibrosis (*n* = 5). (K–N) Representative western blot images and relative expression of COL1A1, COL3A1 and α‐SMA in each group of atria (*n* = 4). Data were expressed as mean ± SEM. **p < 0.01, ***p < 0.001, versus TAC + MAT group; ^ns^ *p* > 0.05, ^###^ *p* < 0.001, versus TAC group. ns, not significant.

To further investigate whether the inhibition of the Wnt3a/β‐catenin signalling pathway contributes to the protective effect of MAT on TAC mice, we utilised SKL2001 to inhibit the phosphorylation of β‐catenin, thereby stabilising and activating its expression [29]. Firstly, we assessed left ventricular function and atrial structure in mice. The results revealed that compared to the TAC + MAT group, cardiac function was significantly impaired and atrial enlargement occurred in the TAC + MAT + SKL2001 group, indicating an abrogation of MAT's protective effect on cardiac function in TAC mice (Figure 5B–H). Additionally, atrial fibrosis levels and the expression of fibrosis‐related markers (COL1A1, COL3A1 and α‐SMA) were assessed using Masson's trichromatic staining and western blotting. The results demonstrated that compared to the TAC + MAT group, the TAC + MAT + SKL2001 group exhibited increased atrial fibrosis levels (Figure 5I,J), as well as elevated expression levels of COL1A1, COL3A1 and α‐SMA proteins (Figure 5K–N). These results suggest that MAT's protective effects on atrial remodelling and cardiac function are associated with inhibition of the Wnt3a/β‐catenin signalling.

### 3.6 Activation of the β‐Catenin Signalling Attenuated the Protective Effect of MAT on Atrial Electrical Signal Conduction

**FIGURE 6:**
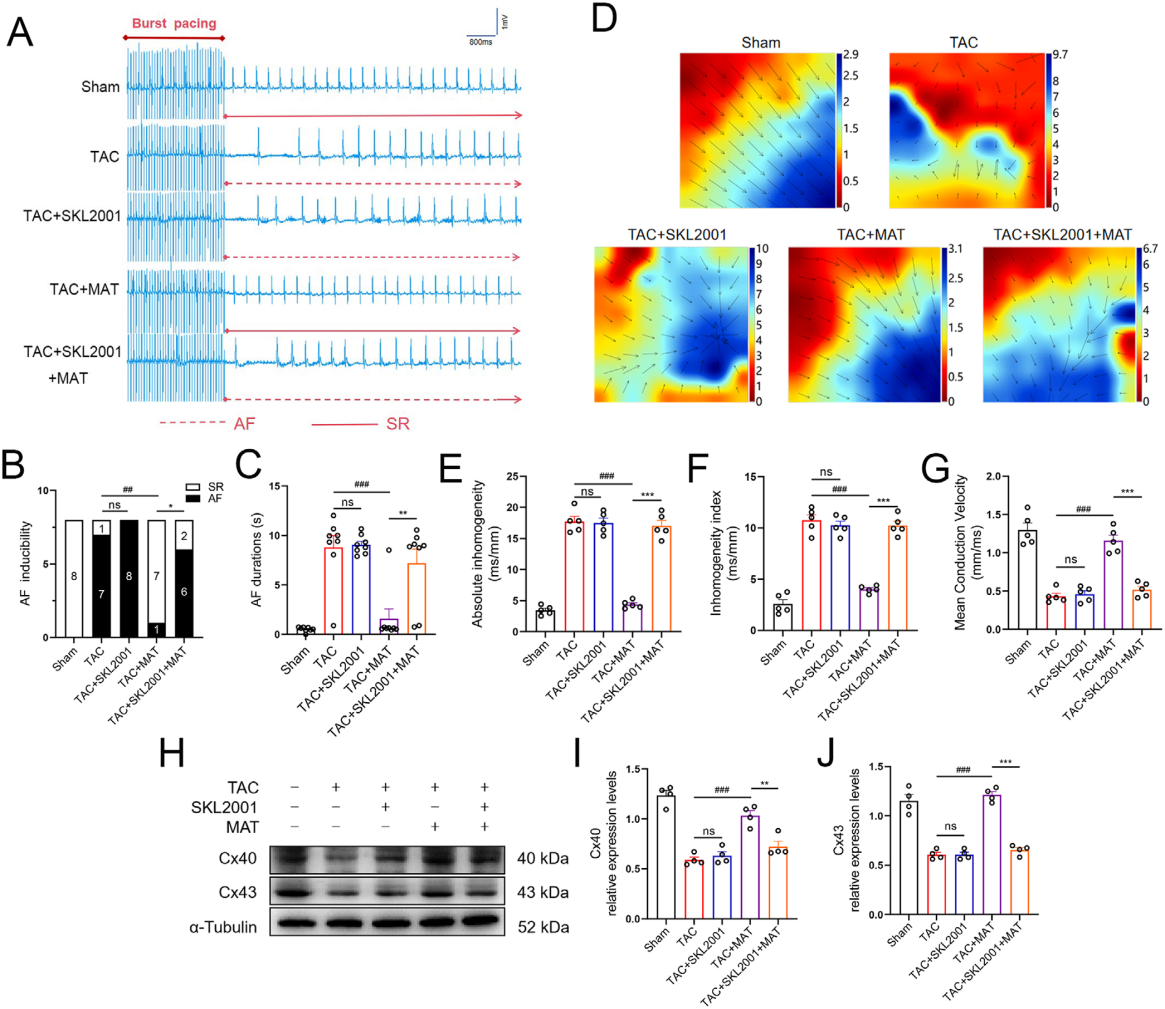
Reversal of the beneficial impact of MAT on atrial electrical remodelling in TAC mice by activation of β‐catenin. (A) Representative ECG traces following burst pacing in the right atrium. The dashed line represents the atrial fibrillation rhythm. (B, C) Summary of AF induction and AF duration in each group (*n* = 8). (D) Langendorff electrophysiological mapping images. The bars represent the duration from the initial to the final measurement within one heartbeat (*n* = 5). (E–G) Summary of left atrial activation patterns. EMapScope 5.0 software was used to calculate the mean conduction velocity, absolute inhomogeneity and inhomogeneity index of the left atrium (*n* = 5). (H–J) Representative western blot images and relative atrial expression of Cx40 and Cx43 (*n* = 4). Data were expressed as mean ± SEM. **p < 0.01, ***p < 0.001, versus TAC + MAT group; ^ns^ *p* > 0.05, ^###^ *p* < 0.001, versus TAC group. ns, not significant.

Next, we investigated the susceptibility of five groups of mice to atrial fibrillation (AF). The results demonstrated that SKL2001 intervention abolished the inhibitory effect of MAT on AF susceptibility, leading to a significant increase in AF susceptibility and a prolonged AF duration in the TAC + MAT + SKL2001 group compared with the TAC + MAT group (Figure 6A–C). Simultaneously, we utilised the Langendorff perfusion system to examine atrial electrical signal conduction, and our findings indicated that MAT can inhibit TAC‐induced atrial electrical remodelling. Nevertheless, these advantageous effects of MAT were negated following the administration of SKL2001(Figure 6D–G). Furthermore, western blot analysis demonstrated a decrease in connexin expression in mice atrial tissue from the TAC + MAT + SKL2001 group compared with the TAC + MAT group, indicating that SKL2001 eliminated MAT's protective effect on gap junction channels (Figure 6H–J). These findings suggest a correlation between Wnt3a/β‐catenin signalling and the inhibitory effect of MAT on atrial remodelling.

### 3.7 Activation of the β‐Catenin Signalling Attenuated the Ameliorative Effects of MAT on Atrial Inflammation and Fibrosis

**FIGURE 7:**
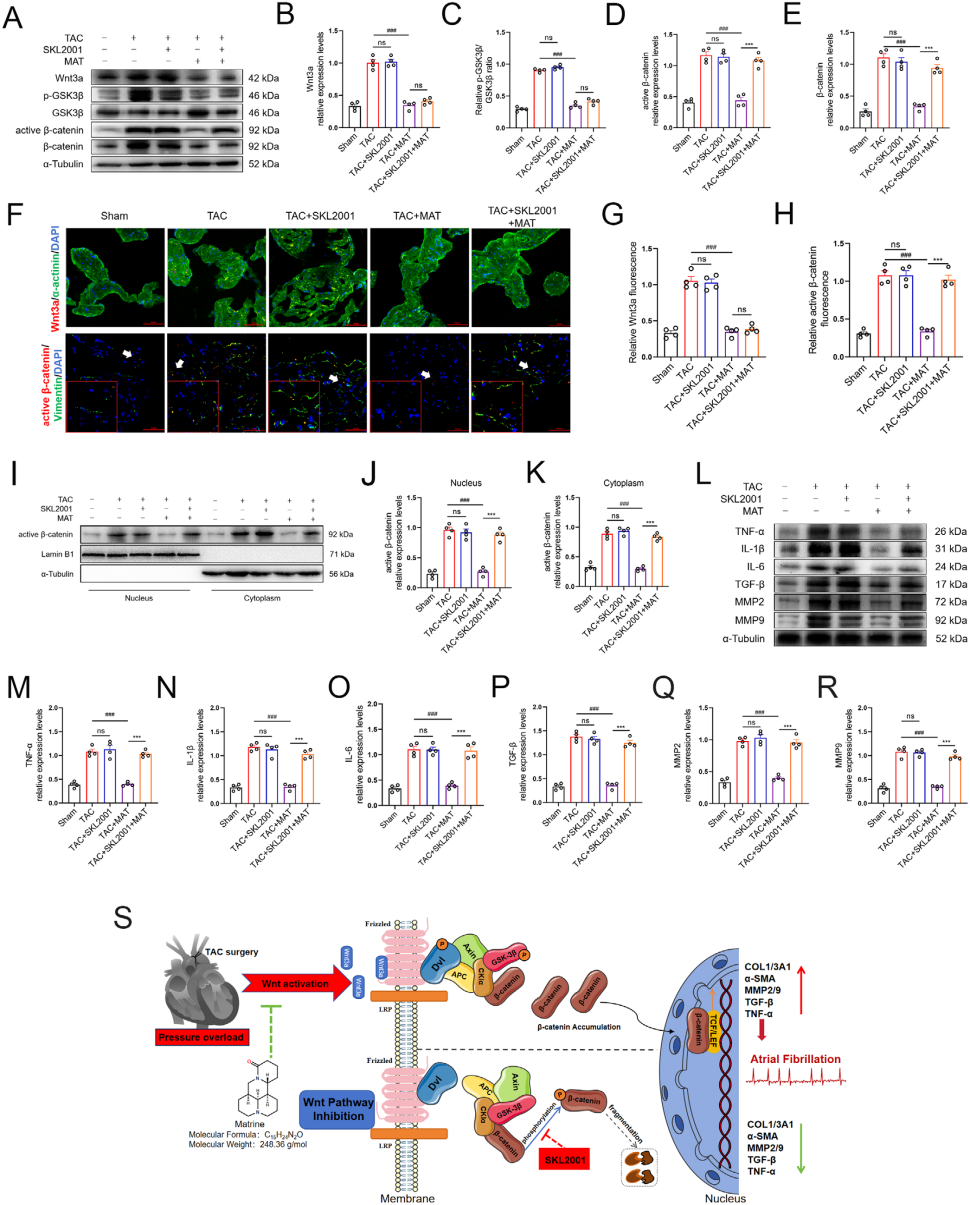
The impact of SKL2001 on the Wnt3a/β‐catenin signalling pathway, atrial fibrosis and inflammation and the mechanisms by which MAT prevents and treats AF. (A–E) Representative western blot images and relative atrial expression of Wnt3a, p‐GSK3β, active β‐catenin and β‐catenin in each group of atria. (F–H) α‐actinin/Vimentin (green), Wnt3a/active β‐catenin (red) and DAPI (blue) immunofluorescence stain representation and Wnt3a/active β‐catenin relative immunofluorescence intensity (*n* = 4). (I–K) Representative western blot images and quantification of active β‐catenin expression in the atrial nucleus or cytoplasm (*n* = 4). (L–R) Representative western blot images and relative atrial expression of TNF‐α, IL‐1β, IL‐6, TGF‐β, MMP2 and MM9. (S) Schematic diagram illustrating the mechanism by which MAT inhibits AF via the Wnt3a/β‐catenin signalling pathway. Data were expressed as mean ± SEM. *n* = 4 per group. ^ns^ *p* > 0.05, ***p < 0.001, versus TAC + MAT group; ^ns^ *p* > 0.05, ^###^ *p* < 0.001, versus TAC group. ns, not significant.

Following treatment with SKL2001, we assessed the expression levels of key molecules involved in the Wnt3a/β‐catenin signalling pathway. Our results revealed a significant increase in active β‐catenin and total β‐catenin expression in the TAC + SKL2001 + MAT group compared to the TAC + MAT group. However, there were no notable differences observed in the expression levels of Wnt3a, p‐GSK3β and GSK3β between these two groups (Figure 7A–I). Immunofluorescence analysis also confirmed these findings for Wnt3a and active β‐catenin (Figure 7F–H). The nuclear and cytoplasmic levels of active β‐catenin were found to be significantly elevated in the TAC + SKL2001 + MAT group compared to the TAC + MAT group (Figure 7I–K). Furthermore, using western blot analysis, we detected significantly elevated levels of TGF‐β, MMP2, MMP9, TNF‐α, IL‐1β and IL‐6 proteins in the TAC + MAT + SKL2001 group compared to the TAC + MAT group (Figure 7L–R). As illustrated in Figure 7S, we elucidated the mechanism by which MAT prevents and treats AF through the Wnt3a/β‐catenin signalling pathway. In summary, our experiments demonstrated that the combination of MAT and SKL2001 counteracted the inhibitory effect of MAT on the Wnt3a/β‐catenin signalling pathway, resulting in atrial fibrosis and inflammation.

## 4 Discussion

In this study, we showed that TAC surgery induces atrial electrical and structural remodelling, leading to a significant increase in susceptibility to AF. Furthermore, bioinformatics analysis unveiled the activation of diverse signalling pathways in the atrial tissue of TAC mice, notably including the canonical Wnt signalling pathway. Subsequent analysis of human heart tissue samples revealed the activation of the Wnt3a/β‐catenin signalling pathway in AF patients, which contributes to fibrotic changes such as collagen deposition within the atrial tissue. Moreover, by integrating network pharmacology and experimental investigations, we have uncovered that a natural compound, MAT, possesses the capability to inhibit the Wnt3a/β‐catenin signalling pathway, thereby attenuating atrial fibrosis and inflammatory responses, ultimately reducing the incidence of TAC‐induced AF (Figure 7S). These findings suggest that MAT demonstrates anti‐arrhythmic effects in pressure overload conditions and underscore the Wnt3a/β‐catenin signalling pathway as a promising target for managing AF associated with pressure overload.

The occurrence of AF is frequently accompanied by a myriad of cardiovascular and cerebrovascular complications, including stroke and HF, thereby posing a significant threat to the lives of affected patients [23, 30]. Therefore, there is an increasing emphasis on elucidating the molecular mechanisms underlying AF. Previously, chronic inflammation and atrial fibrosis have been established as key pathogenic factors in AF [1]. Concurrently, several studies have demonstrated that cardiac pressure overload markedly exacerbates atrial remodelling and fibrosis, thereby fostering an environment conducive to the development of AF [31, 32]. The involvement of the Wnt signalling pathway in cardiac fibrosis has been observed in studies on arrhythmia [33]. The Wnt signalling pathway can be categorised into the canonical and non‐canonical pathways. In the canonical pathway, binding of Wnt protein to its membrane receptor triggers activation of Dishevelled (Dvl), leading to dissociation of a complex comprising casein kinase 1α (CK1α), GSK‐3β, adenomatous colon polyp (APC) scaffold protein and Axin. This process avoids β‐catenin degradation in the cytoplasm, facilitating its nuclear translocation and subsequent binding to T‐cell factor/lymphoid enhancer‐binding factor (TCF/LEF) family transcription factors, thereby initiating downstream target gene transcription and inducing subsequent cellular responses [34]. Wnt3a is a principal signalling protein within the Wnt family and the Wnt3a/β‐catenin signalling pathway has been implicated in arrhythmia diseases. Studies by Cai et al. demonstrated that pathway activation in iPSC‐CM cell models of Brugada syndrome led to reduced Nav1.5 expression, significantly increasing arrhythmia incidence [35]. Additionally, Lv et al. observed activation of the Wnt3a/β‐catenin signalling pathway in Ach‐CaCl_2_ induced AF rats, leading to alterations in collagen levels and gap connexin proteins within atrial tissue, thereby promoting atrial fibrosis and facilitating AF occurrence [36]. Furthermore, previous studies have demonstrated the modulation of TNF‐α levels by Wnt signalling, which forms a positive feedback loop between the two pathways [37]. In line with previous studies, we have also observed a close association between the Wnt3a/β‐catenin signalling pathway and AF, resulting in alterations in atrial fibrosis and inflammatory response. What distinguishes our study is the novel evidence linking AF to the Wnt3a/β‐catenin signalling pathway under pressure overload conditions, offering promise as a potential target for preventing and treating pressure overload‐induced AF.

The process of tissue fibrosis mediated by the Wnt3a/β‐catenin signalling pathway is intricately associated with fibroblasts. Previous studies have demonstrated that overexpression of Wnt3a in fibrocytes leads to a significant upregulation of downstream proteins, including β‐catenin and cyclinD1, resulting in enhanced proliferation of fibroblasts and fibrotic changes. These findings suggest a potential role for this pathway in the development of epidural fibrosis [38]. The presence of similar cascades has been demonstrated in fibroblasts across various studies, including those conducted on kidney and lung tissues [39, 40]. Notably, it has been observed that pressure overload in the right heart resulted in upregulation of the Wnt/ β‐catenin signalling pathway. Stimulation of cardiac fibroblasts with Wnt3a significantly enhanced β‐catenin activation within these cells, facilitating its translocation into the nucleus and subsequently mediating FOS‐proto‐oncogene‐like 1/2 (FOSL1/2) transcriptionally. This cascade ultimately leads to proliferation and aggregation of cardiac fibroblasts, contributing to cardiac remodelling [9]. In addition, in our study, we found that the inflammatory response is downstream of the Wnt3a/β‐catenin pathway and plays an independent role. Other studies have also reported that β‐catenin can enhance its transcriptional activity by directly binding to the NF‐κB promoter region, thereby promoting the release of pro‐inflammatory factors such as TNF‐α and IL‐1 [10]. Notably, the contribution of inflammation to AF extends beyond structural remodelling. It was also observed that MAT treatment significantly restored the expression of Cx40 and Cx43, suggesting that inflammation may damage gap junction proteins through oxidative stress [41], thereby disrupting electrical conduction synchronisation. Clinical studies further revealed that the IL‐1β level in the atrial tissue of patients with AF is positively correlated with the degree of electrical remodelling [42]. Therefore, targeting the Wnt3a/β‐catenin signalling pathway and exploring innovative approaches to modulate this pathway may provide effective strategies for managing AF induced by pressure overload.

The beneficial effects of MAT on the cardiovascular system have been extensively documented in various cardiovascular diseases, including HF, diabetic cardiomyopathy and myocardial ischemia–reperfusion injury [43, 44, 45]. In the context of arrhythmia‐related disorders, MAT can also mitigate the incidence of arrhythmias by modulating ion channels and inhibiting cardiac fibrosis [15, 20]. In this study, we observed that MAT effectively mitigated atrial Wnt3a/β‐catenin signalling and fibrosis markers under conditions of pressure overload. Furthermore, MAT demonstrated the ability to ameliorate atrial electrical and structural remodelling induced by TAC, thereby reducing susceptibility to AF. To our knowledge, this study is the first to unveil the advantageous effects of MAT on pressure overload‐related AF, despite previous reports highlighting its anti‐arrhythmic properties in rat models of myocardial infarction (MI) [20]. Notably, Shi et al. demonstrated that MAT effectively reverses liver epithelial‐mesenchymal transformation (EMT) by inhibiting the Wnt‐1/β‐catenin signalling pathway, thereby suppressing liver fibrosis progression [46]. Moreover, in the investigation of acute myeloid leukaemia (AML), MAT exhibited enhanced induction of apoptosis and cell cycle arrest through inhibition of the Wnt/β‐catenin signalling pathway, thereby exerting a favourable impact on impeding AML progression [47]. Here, we employed a pharmacological intervention to achieve stable expression of β‐catenin in vivo. Remarkably, our findings revealed that the cardioprotective effects of MAT were abrogated upon stable expression of β‐catenin, thereby further substantiating the involvement of the Wnt3a/β‐catenin signalling pathway in mediating MAT's actions.

The study has several limitations. First, our investigation focused on the regulatory effects of Wnt3a on downstream molecules in fibroblasts; however, the potential roles of Wnt3a in other atrial cell types remain largely unexplored. Second, previous studies have demonstrated that Wnt3a is not only closely associated with cardiac fibrosis but also that its upregulation is linked to myocardial hypertrophy [48]. Given the therapeutic efficacy of MAT on ventricular hypertrophy [49], further exploration of its underlying mechanisms is warranted. Finally, other members of the Wnt family have been shown to significantly influence AF pathogenesis. For instance, Feng et al. reported that Ach‐CaCl_2_ stimulation induces upregulation of atrial Wnt5a expression, elevates TGF‐β levels and exacerbates atrial tissue fibrosis, thereby markedly increasing the incidence of AF [50]. These findings strongly suggest that other Wnt family members may also contribute to pressure overload‐induced AF, necessitating further investigation into the therapeutic effects of MAT on these pathways.

## 5 Conclusion

In conclusion, this study reveals that pressure overload triggers activation of the atrial Wnt3a/β‐catenin signalling pathway, resulting in increased susceptibility to AF by fostering atrial fibrosis and inflammatory responses and exacerbating atrial structural and electrical remodelling. Treatment with MAT shows promise in alleviating atrial structural and electrical remodelling by inhibiting the Wnt3a/β‐catenin signalling pathway, consequently lowering the risk of AF development. In summary, our findings underscore the potential of MAT therapy as an intervention to prevent arrhythmias induced by heart failure, offering a promising avenue for managing these cardiac conditions.

## Author Contributions

**Guoxin Zhang:** data curation (equal), formal analysis (equal), writing – original draft (equal), writing – review and editing (equal). **Xue Dong:** visualization (equal), writing – review and editing (equal). **Boxuan Sun:** investigation (equal), methodology (equal). **Zijun Zhou:** investigation (equal), methodology (equal). **Yinli Xu:** investigation (equal), methodology (equal). **Yuting Huang:** investigation (equal), methodology (equal). **Shan Meng:** investigation (equal), methodology (equal). **Zijun Cao:** investigation (equal), methodology (equal). **Nana Qin:** investigation (equal), methodology (equal). **Yan Zhu:** methodology (equal), resources (equal). **Liming Yu:** conceptualization (equal), funding acquisition (equal), project administration (equal), supervision (equal), writing – review and editing (equal). **Huishan Wang:** conceptualization (equal), funding acquisition (equal), project administration (equal), supervision (equal), writing – review and editing (equal).

## Conflicts of Interest

The authors declare no conflicts of interest.

## Supporting information


**Figure S1.** Preliminary analysis of mice in the Sham and Sham+MAT groups. (A) Representative.

**Table S1.** Patient demographics.

**Table S2.** Antibodies information.

**Table S3.** The baseline characteristics of the mice.

## Data Availability

The data supporting the findings of this study are available from the corresponding author upon reasonable request.
