# Supplementary material for: Matrine Inhibits the Wnt3a/β‐Catenin Signalling to Attenuate Pressure Overload‐Induced Atrial Remodelling and Vulnerability to Atrial Fibrillation
Source: J Cell Mol Med. 2025 May 23;29(10):e70617. doi: 10.1111/jcmm.70617 (PMC12101071; doi:10.1111/jcmm.70617)
Supplement: Supplementary file 1 — Figure S1. Preliminary analysis of mice in the Sham and Sham+MAT groups. (A) Representative. [file JCMM-29-e70617-s003.pdf]

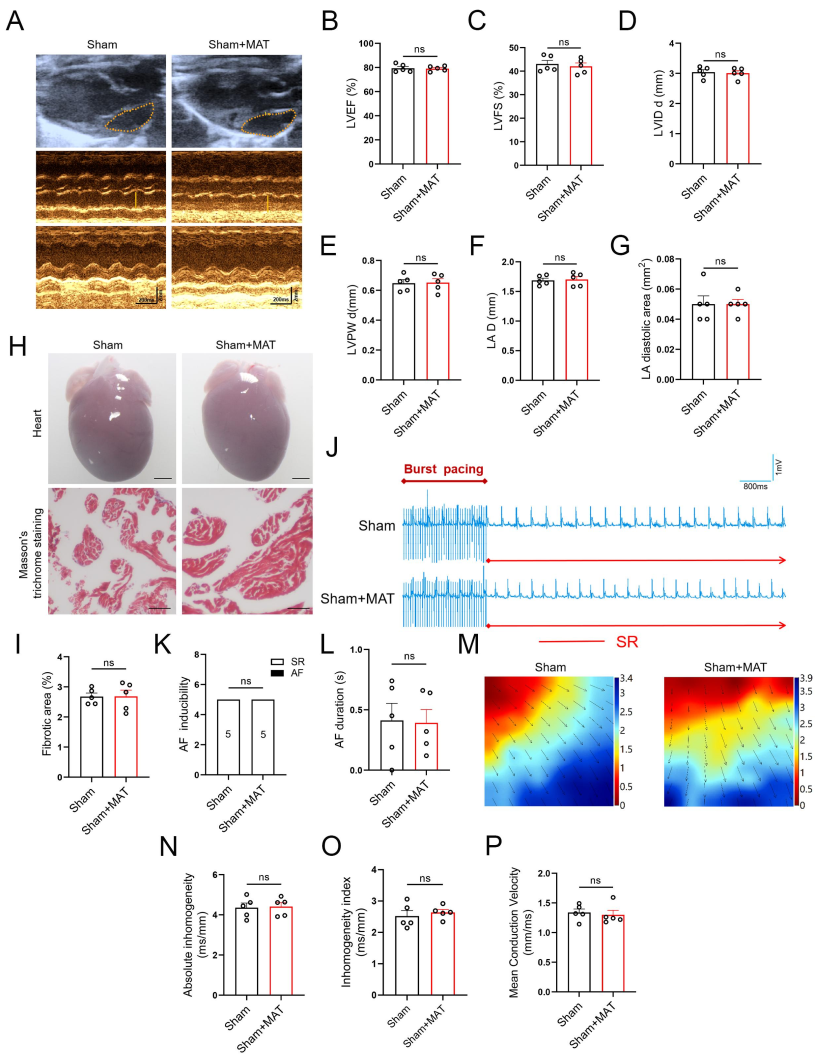

Fig. S1. Preliminary analysis of mice in the Sham and Sham+MAT groups. (A) Representative echocardiographic images of the atria and ventricle. (B-G) Echocardiographic parameters including left ventricular ejection fraction (LVEF), left ventricular shortening fraction (LVFS), left ventricular end-diastolic diameter (LVIDd), left ventricular posterior wall diameter (LVPWd), left atrial diameter (LAD), and left atrial diastolic area. (H) Overall cardiac morphology and representative Masson's trichromatic staining images (upper bar = 2mm, lower bar = 100μm). (I) Percentage of the total area with fibrosis. (J) Representative ECG traces following burst pacing in the right atrium. (K and L) Summary of AF induction and AF duration in each group. (M) Langendorff electrophysiological mapping images. The bars represent the duration from the initial to the final measurement within one heartbeat. (N-P) Summary of left atrial activation patterns. EMapScope 5.0 software was used to calculate the mean conduction velocity, absolute inhomogeneity and inhomogeneity index of the left atrium. Data were expressed as mean ± S.E.M. n = 5 per group. ^{ns}p >0.05, vs. Sham group. ns, not significant.
