# Supplementary material for: Matrine Inhibits the Wnt3a/β‐Catenin Signalling to Attenuate Pressure Overload‐Induced Atrial Remodelling and Vulnerability to Atrial Fibrillation
Source: J Cell Mol Med. 2025 May 23;29(10):e70617. doi: 10.1111/jcmm.70617 (PMC12101071; doi:10.1111/jcmm.70617)
Supplement: Supplementary file 2 — Table S1. Patient demographics. [file JCMM-29-e70617-s002.docx]

| **Table S1. Patient demographics** | | | | | |
| --- | --- | --- | --- | --- | --- |
|  | Sex | Age | Tissues | Diagnosis | Index of cardiac structure and function |
| SR1 | male | 63 | Right atrial  appendage | 1. Degenerative valvular disease 1.1 Mitral insufficiency 1.2 Pulmonary hypertension 2. Heart-failure 3. Hypertension | AoD:22,33,31; LAD:42; IVSD:12; LVIDd:52; LVIDs:37; LVPWD:11; RVOTD:24; PA:21; LVEDV:135ml; LVESV:59ml; EF:0.57; FS:0.3 |
| SR2 | male | 45 | Right atrial  appendage | 1. Degenerative valvular disease 1.1 Mitral insufficiency 1.2 Tricuspid insufficiency 2. Heart-failure 3. Hypertension | AoD:20,34,29; LAD:66; IVSD:10; LVIDd:60; LVIDs:42; LVPWD:10; RVOTD:23; PA:23; LVEDV:181ml; LVESV:73ml; EF:0.53; FS:0.3 |
| SR3 | male | 67 | Right atrial  appendage | 1. Degenerative valvular disease 1.1 Mitral insufficiency 1.2 Pulmonary hypertension 2. Heart-failure 3. Type 2 diabetes | AoD:23,37,33; LAD:45; IVSD:11; LVIDd:50; LVIDs:36; LVPWD:11; RVOTD:25; PA:26; LVEDV:118ml; LVESV:53ml; EF:0.55; FS:0.29 |
| SR4 | male | 69 | Right atrial  appendage | 1. Degenerative valvular disease 1.1 Mitral insufficiency 2. Heart-failure 3. Type 2 diabetes | AoD:21,30,26; LAD:39; IVSD:11; LVIDd:48; LVIDs:33; LVPWD:11; RVOTD:18; PA:20; LVEDV:108ml; LVESV:44ml; EF:0.59; FS:0.31 |
| SR5 | male | 67 | Right atrial  appendage | 1. Rheumatic valvular disease 1.1 Mitral stenosis 1.2 Tricuspid insufficiency 2. Heart-failure 3. Type 2 diabetes 4. Hypertension | AoD:20,30,28; LAD:50; IVSD:14; LVIDd:51; LVIDs:36; LVPWD:14; RVOTD:25; PA:32; LVEDV:125ml; LVESV:54ml; EF:0.56; FS:0.29 |
| SR6 | male | 64 | Right atrial  appendage | 1. Rheumatic valvular disease 1.1 Mitral stenosis 1.2 Pulmonary hypertension 2. Heart-failure 3. Hypertension | AoD:23,36,31; LAD:37; IVSD:9; LVIDd:48; LVIDs:34; LVPWD:9; RVOTD:48; PA:34; LVEDV:110ml; LVESV:48ml; EF:0.57; FS:0.3 |
| AF1 | male | 53 | Right atrial  appendage | 1.Degenerative valvular disease 1.1 Mitral valve stenosis and insufficiency 1.2 Tricuspid insufficiency 2. Atrial fibrillation 3. Heart-failure 4. Hypertension | AoD:23,35,31; LAD:40; IVSD:11; LVIDd:50; LVIDs:35; LVPWD:11; RVOTD:24; PA:24; LVEDV:117ml; LVESV:50ml; EF:0.58; FS:0.3 |
| AF2 | male | 49 | Right atrial  appendage | 1. Degenerative valvular disease 1.1 Mitral valve stenosis and insufficiency 1.2 Tricuspid insufficiency 2. Atrial fibrillation 3. Heart-failure | AoD:20,27,26; LAD:61; IVSD:8; LVIDd:40; LVIDs:29; LVPWD:8; RVOTD:24; PA:38; LVEDV:71ml; LVESV:31ml; EF:0.56; FS:0.29 |
| AF3 | male | 50 | Right atrial  appendage | 1.Degenerative valvular disease 1.1 Mitral valve stenosis and insufficiency 1.2 Tricuspid insufficiency 2. Atrial fibrillation 3. Congestive heart-failure 4. Pulmonary hypertension | AoD:22,35,30; LAD:54; IVSD:10; LVIDd:47; LVIDs:32; LVPWD:10; RVOTD:26; PA:22; LVEDV:99ml; LVESV:41ml; EF:0.59; FS:0.31 |
| AF4 | male | 59 | Right atrial  appendage | 1. Rheumatic valvular disease 1.1 Mitral stenosis 1.2 Dilated tricuspid ring with incomplete closure 2. Atrial fibrillation 3. Congestive heart-failure | AoD:21,34,27; LAD:49; IVSD:10; LVIDd:38; LVIDs:29; LVPWD:10; RVOTD:10; PA:19; LVEDV:64ml; LVESV:31ml; EF:0.53; FS:0.26 |
| AF5 | male | 66 | Right atrial  appendage | 1. Degenerative valvular disease 1.1 Mitral insufficiency 1.2 Tricuspid insufficiency 1.3 Pulmonary hypertension 2. Atrial fibrillation 3. Hypertension 4. Congestive heart-failure | AoD:22,32,28; LAD:63; IVSD:12; LVIDd:61; LVIDs:45; LVPWD:12; RVOTD:31; PA:20; LVEDV:190ml; LVESV:93ml; EF:0.50; FS:0.26 |
| AF6 | male | 55 | Right atrial  appendage | 1. Degenerative valvular disease 1.1 Mitral insufficiency 1.2 Tricuspid insufficiency 2. Atrial fibrillation 3. Heart-failure | AoD:22,32,29; LAD:48; IVSD:11; LVIDd:51; LVIDs:36; LVPWD:11; RVOTD:28; PA:21; LVEDV:126ml; LVESV:55ml; EF:0.57; FS:0.29 |
| Abbreviations: SR, sinus rhythm; AF, atrial fibrillation; AoD, aortic diameter; LAD, left atrium diameter; IVSD, interventricular septal thickness at diastole; LVIDd, left ventricular internal diameter at end-diastole; LVIDs, left ventricular internal diameter at end-systole; LVPWD, left ventricular posterior wall dimension; RVOTD, right ventricular outflow tract diameter; PA, pulmonary artery; LVEDV, left ventricular end-diastolic volume; LVESV, left ventricular end-systolic volume; EF, ejection fraction; FS, fractional shortening. | | | | | |
