# Supplementary material for: Matrine Inhibits the Wnt3a/β‐Catenin Signalling to Attenuate Pressure Overload‐Induced Atrial Remodelling and Vulnerability to Atrial Fibrillation
Source: J Cell Mol Med. 2025 May 23;29(10):e70617. doi: 10.1111/jcmm.70617 (PMC12101071; doi:10.1111/jcmm.70617)
Supplement: Supplementary file 3 — Table S2. Antibodies information. [file JCMM-29-e70617-s004.docx]

**Table S2. Antibodies information**

| **Target**  **antigen** | **Catalog#** | **Vendor**  **or source** | **Working**  **concentration** | **Persistent lD/URL** |
| --- | --- | --- | --- | --- |
| Wnt3a | 26744-1-AP | Proteintech | WB 1:1000 | https://www.ptgcn.com/products/WNT3A-Antibody-26744-1-AP.htm |
| Wnt3a | ab219412 | Abcam | IF 1:200 | https://www.abcam.cn/products/primary-antibodies/wnt3a-antibody-epr21889-ab219412.html |
| active β-catenin | 8814S | Cell Signaling Technology | WB 1:1000  IF 1:200 | https://www.cellsignal.cn/products/primary-antibodies/non-phospho-active-b-catenin-ser33-37-thr41-d13a1-rabbit-mab/8814 |
| β-catenin | 51067-2-AP | Proteintech | WB 1:1000 | https://www.ptgcn.com/products/b-cat-Antibody-51067-2-AP.htm |
| p-GSK3β | 9323S | Cell Signaling Technology | WB 1:1000 | https://www.cellsignal.cn/products/primary-antibodies/phospho-gsk-3b-ser9-5b3-rabbit-mab/9323 |
| GSK3β | 22104-1-AP | Proteintech | WB 1:1000 | https://www.ptgcn.com/products/GSK3B-Antibody-22104-1-AP.htm |
| Connexin 40 | 36-4900 | Invitrogen | WB 1:1000  IF 1:200 | https://www.thermofisher.cn/cn/zh/antibody/product/Connexin-40-Antibody-Polyclonal/36-4900 |
| Connexin 43 | sc-271837 | Santa Cruz Biotechnology | WB 1:500 | https://www.scbt.com/zh/p/connexin-43-antibody-f-7 |
| Connexin 43 | 3512S | Cell Signaling Technology | IF 1:200 | https://www.cellsignal.cn/products/primary-antibodies/connexin-43-antibody/3512 |
| COL1A1 | sc-293182 | Santa Cruz Biotechnology | WB 1:500 | https://www.scbt.com/zh/p/col1a1-antibody-3g3 |
| COL13A | GB111323-100 | Servicebio | WB 1:1000 | https://www.servicebio.cn/goodsdetail?id=3675 |
| α-SMA | 48938S | Cell Signaling Technology | WB 1:1000  IF 1:200 | https://www.cellsignal.cn/products/primary-antibodies/a-smooth-muscle-actin-1a4-mouse-mab-if-formulated/48938 |
| TNF-α | sc-52746 | Santa Cruz Biotechnology | WB 1:500 | https://www.scbio.cn/zh/p/tnfalpha-antibody-52b83 |
| IL-1β | sc-52012 | Santa Cruz Biotechnology | WB 1:500 | https://www.scbio.cn/p/il-1beta-antibody-11e5 |
| IL-6 | 12912S | Cell Signaling Technology | WB 1:1000 | https://www.cellsignal.cn/products/primary-antibodies/il-6-d5w4v-xp-rabbit-mab/12912 |
| TGF-β | sc-130348 | Santa Cruz Biotechnology | WB 1:500 | https://www.scbio.cn/zh/p/tgf-beta1-antibody-3c11 |
| MMP2 | 40994S | Cell Signaling Technology | WB 1:1000 | https://www.cellsignal.cn/products/primary-antibodies/mmp-2-d4m2n-rabbit-mab/40994 |
| MMP9 | sc-13520 | Santa Cruz Biotechnology | WB 1:500 | https://www.scbio.cn/zh/p/mmp-9-antibody-7-11c |
| α-Tubulin | GB15201-100 | Servicebio | WB 1:1000 | https://www.servicebio.cn/goodsdetail?id=11602 |
| Vimentin | 60330-1-lg | Proteintech | IF 1:200 | https://www.ptgcn.com/products/Vimentin-Antibody-60330-1-Ig.htm |
| Lamin B1 | ab133741 | Abcam | WB 1:1000 | https://www.abcam.cn/products/primary-antibodies/lamin-b1-antibody-epr8985b-nuclear-envelope-marker-ab133741.html |
