# Supplementary material for: Matrine Inhibits the Wnt3a/β‐Catenin Signalling to Attenuate Pressure Overload‐Induced Atrial Remodelling and Vulnerability to Atrial Fibrillation
Source: J Cell Mol Med. 2025 May 23;29(10):e70617. doi: 10.1111/jcmm.70617 (PMC12101071; doi:10.1111/jcmm.70617)
Supplement: Supplementary file 4 — Table S3. The baseline characteristics of the mice. [file JCMM-29-e70617-s001.docx]

**Table S3. The baseline characteristics of the mice**

| **Baseline feature/**  **Groups** | **Gain Weight (g)** | **Body Weight (g)** | **Heart Weight (mg)** | **LA Weight (mg)** | **LA weight /Body Weight (%)** | **Fasting Blood Glucose (mmol/L)** | **AF Incidence** | **AF Duration (s)** | **P Wave Width (ms)** |
| --- | --- | --- | --- | --- | --- | --- | --- | --- | --- |
| **Sham** | 4.98 ± 0.49 | 28.92 ± 1.28 | 162.60 ± 7.72 | 8.04 ± 0.59 | 0.028 ± 0.001 | 6.96 ± 0.34 | 0 | 0.12 ± 0.08 | 11.32 ± 0.73 |
| **Sham+MAT** | 4.88 ± 0.38 | 28.82 ± 0.86 | 163.80 ± 6.95 | 7.94 ± 0.70 | 0.027 ± 0.002 | 6.90 ± 0.28 | 0 | 0.14 ± 0.05 | 11.44 ± 0.48 |
| **TAC** | 3.14 ± 0.36 | 25.70 ± 0.63 | 214.60 ± 4.61 | 11.14 ± 0.55 | 0.043 ± 0.002 | 6.86 ± 0.46 | 88% | 8.28 ± 0.75 | 20.08 ± 0.90 |
| **TAC+L-MAT** | 3.86 ± 0.23 | 26.76 ± 0.65 | 194.00 ± 3.23 | 9.40 ± 0.38 | 0.035 ± 0.001 | 6.80 ± 0.56 | 63% | 4.78 ± 1.18 | 15.36 ± 0.92 |
| **TAC+H-MAT** | 4.56 ± 0.40 | 28.88 ± 0.64 | 174.40 ± 4.85 | 8.30 ± 0.46 | 0.029 ± 0.002 | 7.04 ± 0.63 | 25% | 1.06 ± 1.21 | 12.04 ± 0.57 |
| **TAC+**  **SKL2001** | 3.10 ± 0.31 | 25.46 ± 0.62 | 215.30 ± 5.30 | 11.52 ± 0.78 | 0.045 ± 0.002 | 6.76 ± 0.50 | 100% | 8.36 ± 0.88 | 19.88 ± 1.11 |
| **TAC+SKL2001+MAT** | 3.56 ± 0.29 | 26.14 ± 0.32 | 196.80 ± 2.00 | 10.16 ± 0.31 | 0.039 ± 0.002 | 6.92 ± 0.60 | 75% | 5.87 ± 1.31 | 17.24 ± 0.77 |

All data in the table are presented as mean ± SD. n = 5 per group.
